# The CT assessment of uncovertebral joints degeneration in a healthy population

**DOI:** 10.1186/s40001-021-00619-2

**Published:** 2021-12-13

**Authors:** Tianji Huang, Jie Qin, Weiyang Zhong, Ke Tang, Zhengxue Quan

**Affiliations:** Department of Orthopedic Surgery, The First Affiliated Hospital of Chongqing Medical University, Chongqing, China

**Keywords:** Cervical spine, Uncovertebral joints, Degeneration, CT

## Abstract

**Background:**

A retrospective study investigated the degeneration trend of uncovertebral joints in a healthy population based on CT assessment.

**Methods:**

A total of 200 males and 160 females, aged 21–79 years old (50.82 ± 17.06), who underwent CT examination in our hospital from September 2020 to March 2021 were enrolled. Sixty patients were included in each age group. According to the Kellgren and Lawrence classification and CT was used to evaluate the uncovertebral joints degeneration in different groups.

**Results:**

With the increase of age, the degeneration of each segment was gradually aggravated. The uncovertebral joints started degenerating in the 20 s, and the C5–6 is the most degenerative segment, followed by the C4–5 and C6–7. Significant degeneration occurred in each segment between the 40 s and 60 s and became more severe after the 70 s.

**Conclusions:**

The modified Kellgren and Lawrence classification based on CT scan could provide a quantitative assessment of uncovertebral joints degeneration in a healthy population and could provide more details for artificial cervical arthroplasty.

## Introduction

The biomechanical function of the cervical uncovertebral joints is to limit lateral flexion. The studies demonstrated that the uncovertebral joints are posterolateral in the lower cervical spine, and the base width of the uncinate process gradually increases. The uncovertebral joints closed to the upper vertebral body face more stress than other parts of the vertebral body which could stimulate degeneration [1, 2]. Meanwhile, the uncovertebral joint capsule is thin, and it is easy to degenerate if there is excessive activity, strain and bad posture, although the uncovertebral joint degeneration is a slow process [3–5]. Sun et al. [6] first reported a quantitative classification of uncovertebral joint degeneration and correlation with the severity of the heterotopic ossification after cervical artificial disc replacement, using the X-rays. However, there is still no unified standard for quantifying the uncovertebral joint degeneration. X-rays could be affected by many factors such as imaging techniques, machines or individual differences. Although MRI has a good display effect on the joint degeneration, its coronal plane reconstruction is not ideal [6–8]. As a non-invasive examination, CT assessment could provide three-dimensional images, which is great value for evaluating the degeneration. Based on CT examination, the study aimed to observe the trend of the degeneration of the uncovertebral joints in a healthy population.

## Methods

This study was approved by the Institutional Review Board of The First Affiliated Hospital of Chongqing Medical University and was conducted according to the principles of the Declaration of Helsinki. All the patients provided their written informed consent to participate in our study before the storage of their data in the hospital database. Inclusion criteria: aged 20–79 years had neck pain and completed cervical CT three-dimensional (3D) reconstruction in the physical examination center from September 2020 to March 2021 were randomly selected. Exclusion criteria: cervical fracture, primary cervical tumors or metastatic cervical tumors, cervical tuberculosis, or cervical infection.

## Outcome assessment

**Fig. 1:**
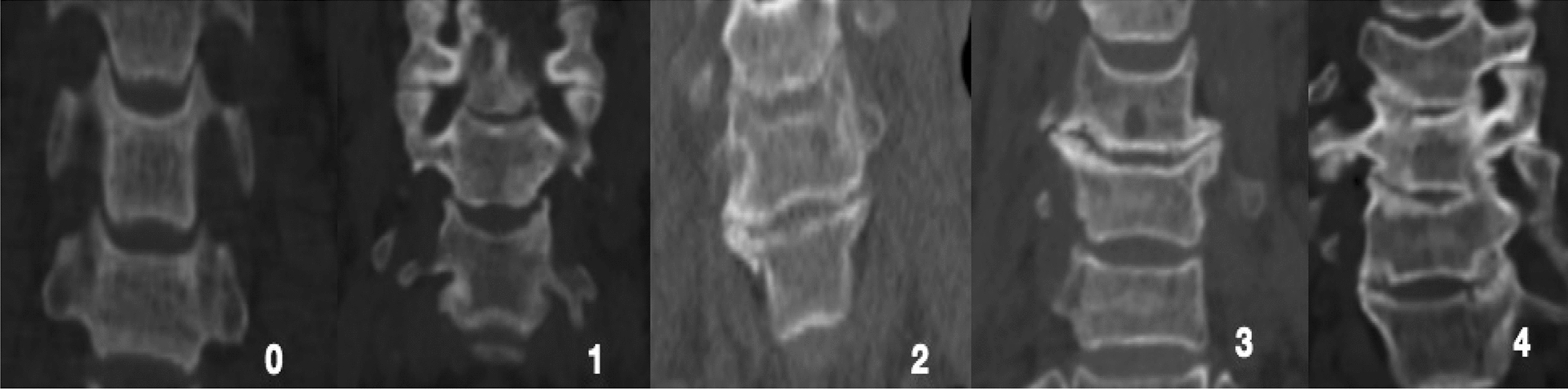
Modified classification

**Fig. 2:**
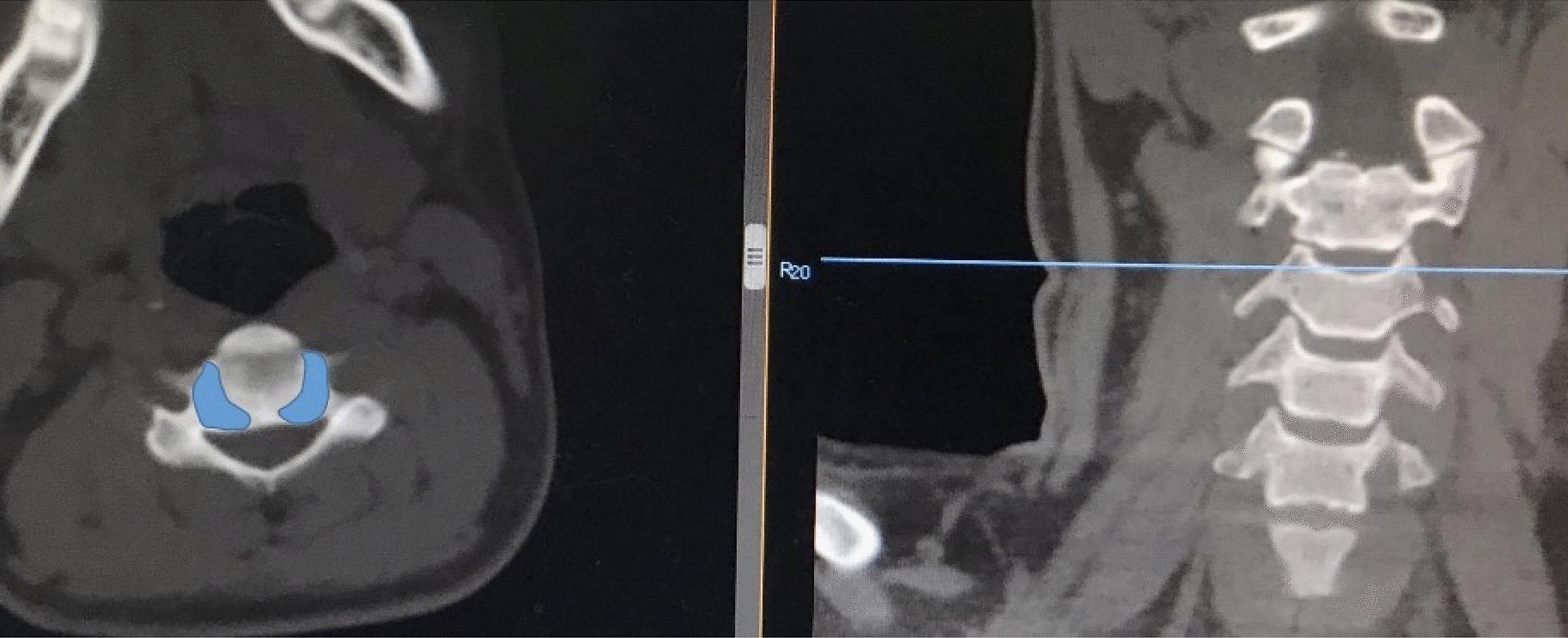
The uncovertebral joints were observed fully on CT

All patients underwent CT scan (Siemens, Germany) and a PACS image system was used for 3D reconstruction. The scan range was selected from C1 to T2 and the thickness of the layer was 0.5 mm. The modified Kellgren and Lawrence classification [9] was assessed for uncovertebral joints degeneration: Grade 0: the joints were normal, bilateral, and symmetrical. Grade 1: mild space narrowing or osteophyte formation. Grade 2: joint space narrowing or osteophyte formation with osteophytes not exceeding the intervertebral level. Grade 3: joint space definite narrowing or osteophyte formation with osteophyte formation exceeding the intervertebral level. Grade 4: osteophyte articulation or uncinate joint fusion (Figs. 1, 2).

## Statistical analysis

The statistic analysis system software (SAS Institute, Inc., Cary, NC, USA) was used for analyzing the data. Quantitative variables are expressed as the mean  ±  SD. The Chi-squared test was used for the categorical variables. The P  < 0.05 was considered statistically significant.

## Results

**Fig. 3:**
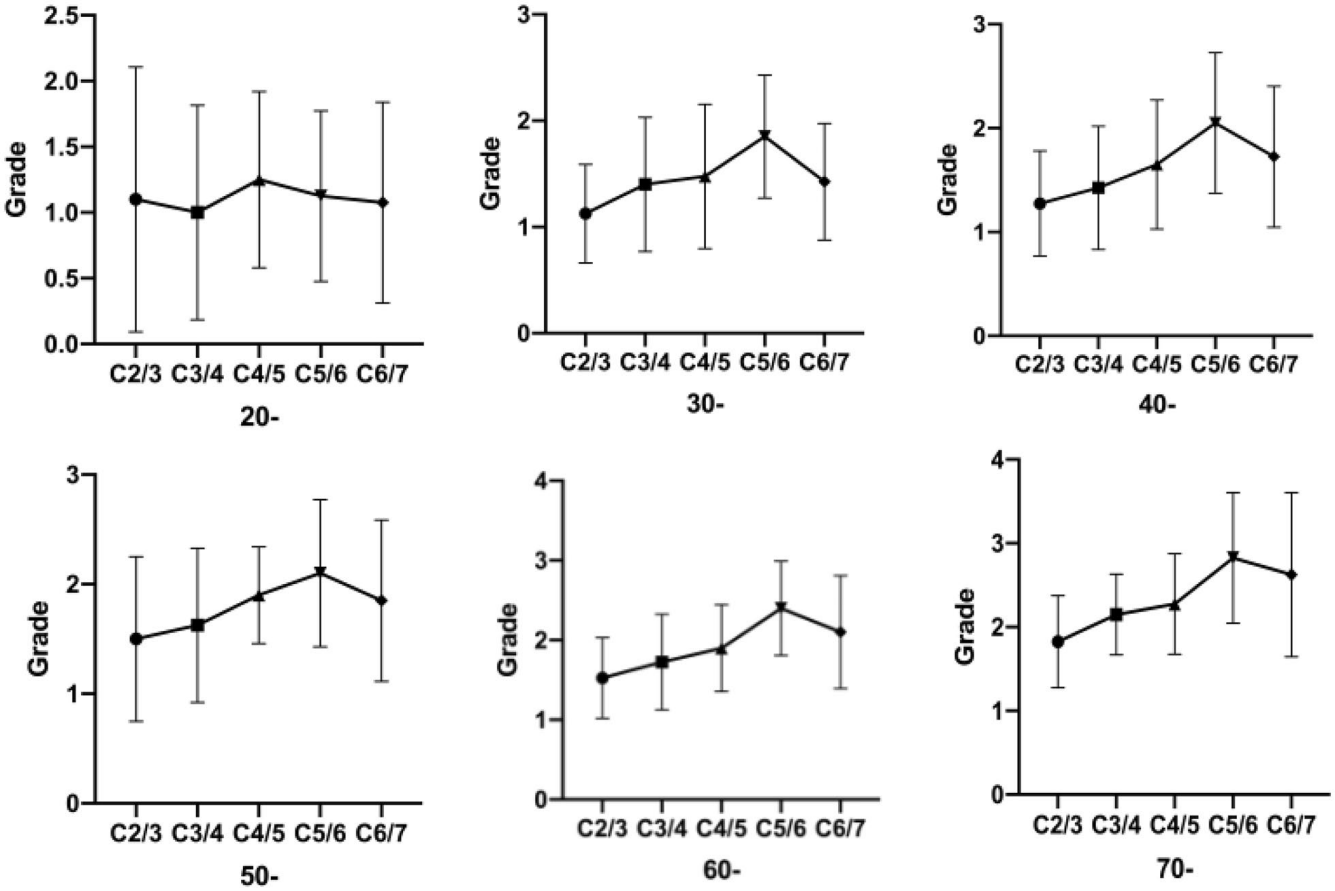
The uncovertebral joints degeneration trend of each segment in different ages

**Fig. 4:**
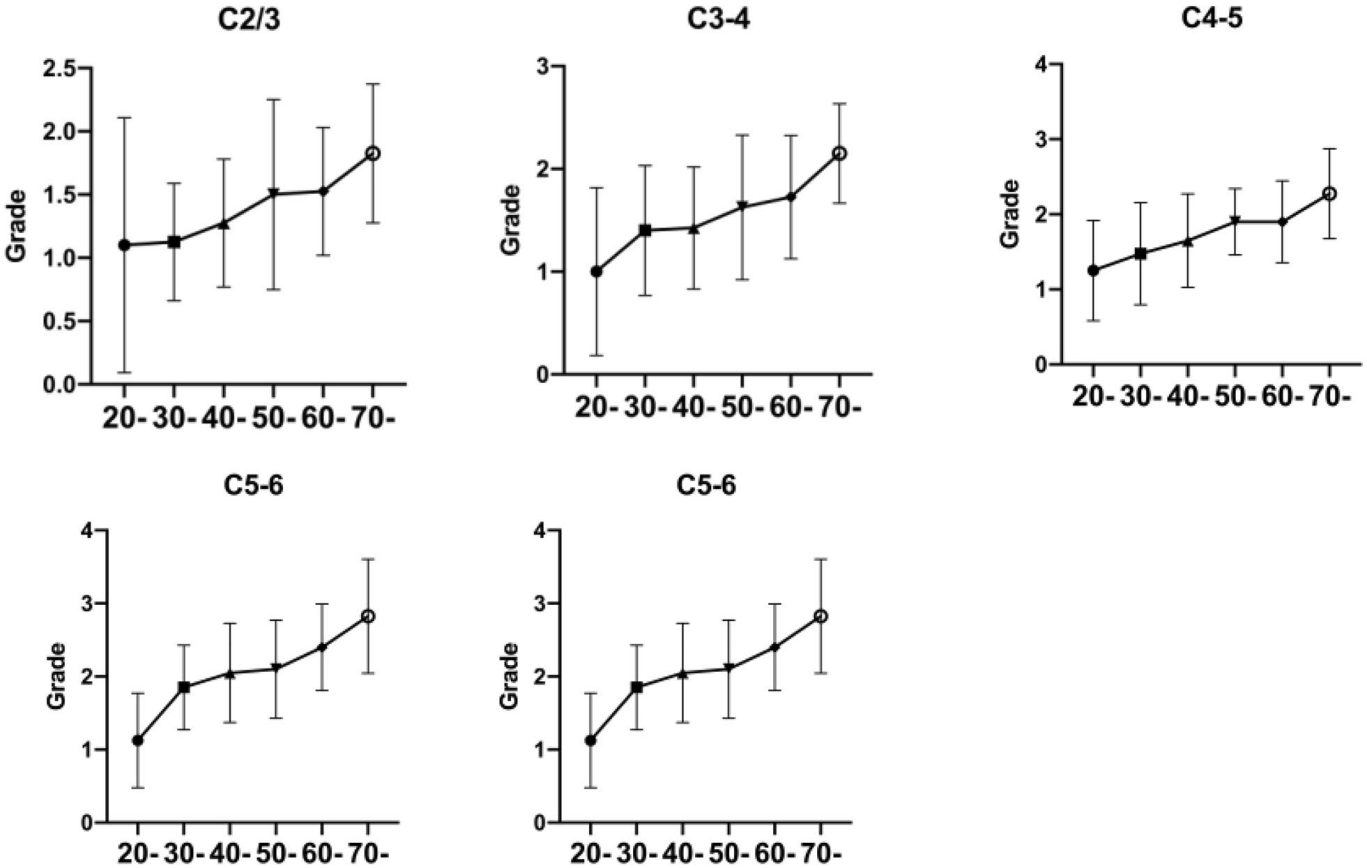
The uncovertebral joints degeneration trend of age in different segments

A total of 360 patients were included, including 200 males and 160 females, aged 21–79 years (50.82 ± 17.06). Sixty patients were included in each age group. With the increase of age, the degeneration of each segment was gradually aggravated (Table 1; Figs. 3,  4). In the group of 20 s, the most severe degeneration was the C4–5 segment, but in other groups, the most severe degeneration was at the C5–6 segment, followed by the C4–5. In the C2–3 and C3–4 segments, there was little change trend of degeneration with the increase of age. However, after the 70 s, the degeneration was significantly more aggravated than before. In the C4–5 segment, with the increase of age, the degeneration accelerated in the 50 s and 70 s, but it degenerated at a slow process in the 60 s. The C5–6 segment starts rapid degeneration from the age of 20 s, while the degeneration trend is relatively stable between 30 and 50 s, while the degeneration trend increases after the 50 s. The degeneration trend of the C6–7 segment is also rapid after the 20 s, while the degeneration trend is relatively slow between the 40 s and 60 s. However, the degeneration is significantly worse after the 70 s.

**Table 1:** The uncovertebral joints degeneration in different groups of age

	20-	30-	40-	50-	60-	70-
C2–3	1.10 ± 1.00	1.13 ± 0.46	1.28 ± 0.51	1.50 ± 0.75	1.55 ± 0.50	1.83 ± 0.55
C3–4	1.00 ± 0.82	1.40 ± 0.63	1.43 ± 0.59	1.63 ± 0.70	1.73 ± 0.60	2.10 ± 0.48
C4–5	1.25 ± 0.67	1.48 ± 0.68	1.65 ± 0.62	1.90 ± 0.44	1.90 ± 0.55	2.28 ± 0.60
C5–6	1.13 ± 0.65	1.85 ± 0.58	2.05 ± 0.68	2.10 ± 0.67	2.40 ± 0.59	2.83 ± 0.78
C6–7	1.08 ± 0.76	1.43 ± 0.55	1.73 ± 0.68	1.85 ± 0.74	2.10 ± 0.71	2.63 ± 0.98

## Discussion

The studies have shown the presence of fibrous cartilage in the uncovertebral joints of the cervical spine, suggesting that the uncovertebral joints as the synovium joint which are closely related to the range of motion. From the perspective of biomechanics, the uncovertebral joints are mainly responsible for the anterior and posterior movement of the vertebral body and the limitation of lateral motion [1–3]. The surgeons are aware of the morphology and adjacent relationship of the uncovertebral joints and its borders the lateral line of the intervertebral disc. The degenerative changes or osteophytes may also occur in uncovertebral joints with the increase of age, resulting in radiculopathy, myelopathy and vertebral vascular insufficiency due to their anatomical relationship with the spinal nerve roots, vertebral artery and spinal cord. The studies reported that the intervertebral disc degeneration included the osteophytes formation and intervertebral space stenosis, and the decrease in height of intervertebral space will directly affect the height of uncovertebral joints. Therefore, it could be concluded the intervertebral disc degeneration is serious while the degeneration of uncinate vertebral joint could be also more severe [4–6].

Few studies focus on the assessment of uncovertebral joints degeneration. Sun et al. [6] firstly quantified the degeneration of uncovertebral joints on X-ray and proposed that there was a positive correlation between the severity of heterotopic ossification (HO) after artificial cervical arthroplasty (ACR) and the severity of preoperative uncovertebral joints degeneration. However, there is still no unified standard for the quantification of uncovertebral joints degeneration. The technique factors of X-ray or individual differences or the imaging experience could affect the judgment of uncovertebral joints degeneration while patients with short necks may fail to judge the degeneration of uncovertebral joints. Although MRI shows the images more clearly and intuitively, its coronal reconstruction is not ideal or the quality of reconstruction images is poor. As a non-invasive scan, CT scan provides 3D images, which is great of value in judging the degeneration of uncovertebral joints. The CT coronal and axial reconstruction at the same time can not only display one level, but also observe the whole structure of uncovertebral joints, and also observe the degeneration of bilateral joints. CT scan is popular in many primary hospitals, and it is easily applied. Sun et al. [6] proposed a method to divide the uncinate joint into three equal parts. However, one of the main biomechanic functions of the uncovertebral joints is to limit the lateral movement of the cervical spine, and the degeneration often starts from the lateral part of the joint [7, 8]. We fully observed the degeneration of cervical uncovertebral joints referred to Kellgren and Lawrence classification and other researches relevant degeneration classification, based on the CT images, proposed a new classification method of evaluating the degeneration of cervical uncovertebral joints, which has strong reliability, easy implementation, good repeatability, and consistency.

It was reported that cervical disc degeneration begins in the 20 s, and the severity of disc degeneration increases with age, among which the C56 disc degeneration is the most serious [9–13]. Our study showed that the uncovertebral joints also started the degeneration in the 20 s and the C5–6 was also most the degenerated segment, followed by the C4–5 and C6–7. Significant degeneration occurred in each segment between the 40 and 60 years and aggravated rapidly after the 70 years. The trend of uncovertebral joints degeneration was consistent with the trend of cervical disc degeneration. Anatomically, the uncovertebral joints restrict lateral motion, and the intervertebral disc degeneration results in changes in intervertebral height which could accelerate uncovertebral joints degeneration. Therefore, the patients with severe intervertebral disc degeneration also have severe uncovertebral joints degeneration which could lead to symptoms [14–16]. Preoperative evaluation grade of the uncovertebral joints of the patients provides more details and references for surgical decision-making and planning.

However, our study had some limitations. First, the study was performed in single-center related with bias. Second, the quantification of uncovertebral joints degeneration is still controversial. In the future, prospective, randomized, multicenter researches with long-term follow-up periods are needed.

## Conclusions

The modified Kellgren and Lawrence classification based on CT scan could provide a quantitative assessment of uncovertebral joints degeneration in a healthy population and could provide more details for artificial cervical arthroplasty.

## Data Availability

The datasets generated and/or analyzed during the current study are not publicly available since the data are confidential patient data, but are available from the corresponding author on reasonable request.

## Abbreviations

CT: Computed tomography
3D: Three-dimensional
HO: Heterotopic ossification
ACR: Artificial cervical arthroplasty

## References

[1] Noh SH, Park JY, Kuh SU (2020). Association of complete uncinate process removal on 2-year assessment of radiologic outcomes: subsidence and sagittal balance in patients receiving one-level anterior cervical discectomy and fusion. BMC Musculoskelet Disord.

[2] Maduri R, Bobinski L, Duff JM (2020). Minimally invasive anterior foraminotomy for cervical radiculopathy: how I do it. Acta Neurochir.

[3] Jong-Uk M, Rae CH, Hwan KS (2019). Uncinate process area as a new sensitive morphological parameter to predict cervical neural foraminal stenosis. Pain Phys.

[4] Kang H, Park P, La Marca F (2010). Analysis of load sharing on uncovertebral and facet joints at the C5–6 level with implantation of the Bryan, Prestige LP, or ProDisc-C cervical disc prosthesis: an in vivo image-based finite element study. Neurosurg Focus.

[5] Chung SB, Muradov JM, Lee SH (2012). Uncovertebral hypertrophy is a significant risk factor for the occurrence of heterotopic ossification after cervical disc replacement: survivorship analysis of Bryan disc for single-level cervical arthroplasty. Acta Neurochir.

[6] Cao S, Pan SF, Sun Y (2020). The correlation between the severity of uncovertebral joints degeneration and heterotopic ossification after single-level artificial cervical disc replacement. Zhonghua Yi Xue Za Zhi.

[7] Tubbs RS, Rompala OJ, Verma K (2012). Analysis of the uncinate processes of the cervical spine: an anatomical study. J Neurosurg Spine.

[8] Kumaresan S, Yoganandan N, Pintar FA (2001). Contribution of disc degeneration to osteophyte formation in the cervical spine: a biomechanical investigation. J Orthop Res.

[9] Kohn MD, Sassoon AA, Fernando ND (2016). Classifications in brief: Kellgren-Lawrence classification of osteoarthritis. Clin Orthop Relat Res.

[10] Dvorak J, Sutter M, Herdmann J (2003). Cervical myelopathy: clinical and neurophysiological evaluation. Eur Spine J.

[11] Lee SH, Son DW, Lee JS (2020). Relationship between endplate defects, modic change, facet joint degeneration, and disc degeneration of cervical spine. Neurospine.

[12] Chen J, Li J, Qiu G (2016). Incidence and risk factors of axial symptoms after cervical disc arthroplasty: a minimum 5-year follow-up study. J Orthop Surg Res.

[13] Wang QL, Tu ZM, Hu P (2020). Long-term results comparing cervical disc arthroplasty to anterior cervical discectomy and fusion: a systematic review and meta-analysis of randomized controlled trials. Orthop Surg.

[14] Pescatori L, Tropeano MP, Visocchi M (2020). Cervical spondylotic myelopathy: when and why the cervical corpectomy?. World Neurosurg.

[15] Moghaddamjou A, Badhiwala JH, Fehlings MG (2020). Degenerative cervical myelopathy: changing frontiers. World Neurosurg.

[16] Wilson JR, Barry S, Fischer DJ (2013). Frequency, timing, and predictors of neurological dysfunction in the nonmyelopathic patient with cervical spinal cord compression, canal stenosis, and/or ossification of the posterior longitudinal ligament. Spine.

